# Clinical and Functional Improvement Following Cariprazine– Olanzapine Combination Therapy in First-Episode Psychosis With Treatment Resistance

**DOI:** 10.1192/bjo.2026.11891

**Published:** 2026-06-30

**Authors:** Neeraja Manoj, Raluca Miutescu, Tynnesia Noel

**Affiliations:** Cygnet Health Care, London, United Kingdom

## Abstract

**Aims:**

First-episode psychosis with treatment resistance can present with diagnostic uncertainty and often necessitate complex psychopharmacological decisions alongside multidisciplinary interventions. Selecting effective antipsychotic treatment while minimising adverse effects is critical, particularly when initial monotherapy is unsuccessful. This case report reviews clinical management and functional improvements in a young woman admitted to an acute psychiatric ward following combination treatment with Cariprazine and Olanzapine, supported by multidisciplinary team interventions.

**Methods:**

X is a young adult female with no prior secondary-care mental health involvement, a family history of schizophrenia. She presented following a 2.5 year deterioration marked by aggression, social decline, and psychotic symptoms. On admission, she was presenting with behavioural disturbance, exhibited prominent paranoia, persecutory delusions, and auditory hallucinations. Initial treatment with oral Olanzapine was limited by non-compliance.

Zuclopenthixol depot was initiated but discontinued due to extrapyramidal side effects. Subsequent treatment with Amisulpride resulted in limited symptom control and cognitive slowing. Clozapine was deferred due to abnormal inflammatory and cardiac markers. A switch and gradual increase to Cariprazine 4.5 mg/d led to an improvement in insight and behaviour, with low-dose Olanzapine 5 mg/d later added to address residual positive symptoms (auditory hallucinations).

MDT input included extensive collateral history, family involvement, and structured Occupational Therapy and Psychology interventions.

**Results:**

This case suggests that Cariprazine augmented with low-dose Olanzapine may be an effective and tolerable strategy in treatment-resistant psychosis when Clozapine is contraindicated. Improvements in functional and psychological outcome measures underscored gradual symptomatic recovery.Symptom severity, measured using the Brief Psychiatric Rating Scale (BPRS), reduced from 38 to 29, representing 23.7 % improvement with the most marked improvement observed in unusual thought content and hallucinations.

The Occupational Therapy Task Observation Scale (OTTOS) - total score improved from 121/200 (08/10/25) to 182/200 (09/01/26), representing a 50.4% increase. Task Behaviour improved by 39.7%, and General Behaviour by 62.1%, with marked gains in engagement, independence, and socialisation.

Autism Spectrum Quotient (AQ-50) score reduced from 22 to 15, a 31.8% reduction in self-reported autistic-type traits. The greatest improvements were observed in Social Skills (90% of items) and Communication (70%), indicating improved interpersonal functioning and cognitive flexibility.

GAP (Global Assessment of Progress) score improved from 29/70 on admission to 63/70, representing an absolute increase of 34 points and reflecting a 48.6% improvement across the different domains.

**Conclusion:**

Combination treatment with Cariprazine and Olanzapine was associated with meaningful symptomatic, functional, and psychological improvement, demonstrating its potential role in complex treatment resistant psychosis.

